# Impact of *Aspergillus* Species on Microbial Community Dynamics and Their Associations with Fermentation Properties in Fermented Walnut-Based Soy Sauce

**DOI:** 10.3390/foods14223921

**Published:** 2025-11-17

**Authors:** Xiaogang Guo, Menghui Lin, Thanh Ninh Le, Zhihong Zhou, Minjie Zhao, Haiying Cai

**Affiliations:** 1 School of Biological and Chemical Engineering, Zhejiang University of Science & Technology, Hangzhou 310023, China; gxg13709097644@163.com (X.G.); 212403817025@zust.edu.cn (M.L.); 2 Department of Food Science and Engineering, National University of Singapore, Singapore 117542, Singapore; itninh90@gmail.com; 3 College of Biosystems Engineering and Food Science, Zhejiang University, Hangzhou 310058, China; zhaominjie@zju.edu.cn; 4 Wushi Xianhong Food Brewing Co., Ltd., Aksu 843400, China; zhihongzhou666@163.com

**Keywords:** *Aspergillus*, walnut-based soy sauce, microbial community, fermentation, walnut polyphenols

## Abstract

This study investigated microbial community dynamics and their links to fermentation traits in solid-state fermentation of walnut -based soy sauce (WSS) using walnut meal-soybean meal mixtures. Via 16S rRNA sequencing and molecular docking, it analyzed the effects of three distinct starter culture treatments—*Aspergillus oryzae* (AO), *Aspergillus niger* (AN), and mixed starter culture (*A. oryzae* + *A. niger*, ON)—as well as fermentation duration on microbial diversity and physicochemical properties, aiming to clarify microbial-driven quality mechanisms. Physicochemical analysis demonstrated superior fermentation performance in the AO group, showing significantly higher amino nitrogen (NH_3_-N) accumulation (0.23 g/100 mL) and protease activity (30.5 U/mL) compared to the AN group, with the mixed inoculation group (ON) exhibiting intermediate results, indicating *A. oryzae*’s dominant role in mixed fermentation. Via PCA and Shannon index, microbial diversity analyses revealed starter cultures shaped microbial community structure: *Enterococcus* and Staphylococcaceae were enriched by AO starter, and *Klebsiella* dominated in AN group. Additionally, temporal succession of the microbiota occurred during post-fermentation of WSS, with Lactobacillales, *Staphylococcus*, and special flavor-producing functional flora dominating early, middle, and later stages, respectively. *Staphylococcus* positively correlated with protease activity and amino nitrogen, critical for quality. Molecular docking showed major walnut polyphenols significantly affected protease activity, aiding process optimization. This research provides theoretical foundations for improving WSS production and enriches understanding of solid-state fermentation microbial ecology.

## 1. Introduction

Solid-state fermentation, as a gem of traditional Chinese food processing techniques, has given rise to a series of fermented products with unique flavors and cultural significance, such as soy sauce, vinegar, and baijiu. Among these, soy sauce stands out as an ideal model for analyzing the microbial ecosystem of solid-state fermentation due to its complex microbial interaction network and rich flavor compounds [1]. The fermentation of soy sauce occurs in two main stages: koji-making and moromi fermentation. Microbial enzymes hydrolyze macromolecules into smaller units, which further undergo biochemical reactions to produce flavor compounds, contributing to the characteristic flavor and quality of soy sauce [2]. In the microbial community of soy sauce fermentation, molds, yeasts, and bacteria work synergistically. *A. oryzae* (AO), as the core microorganism in the koji-making stage, secretes various extracellular enzymes, particularly proteases and amylases, playing a pivotal role in the degradation of proteins and starch in the raw materials [3]. By contrast, *A. niger*, a versatile auxiliary mold widely used in traditional fermented foods, possesses a distinct enzyme system that complements AO’s functions. AN is particularly proficient in secreting glycoside hydrolases, including cellulases, hemicellulases, and pectinases, which can break down complex polysaccharides in plant-based substrates—substances that AO struggles to decompose efficiently. Additionally, AN produces acidic proteases (optimal pH 2.0–5.0) that retain activity in slightly acidic microenvironments, a trait that may adapt to the dynamic pH changes during WSS fermentation [4].

Recently, the demand for functional foods has driven interest in novel soy sauces derived from walnut meal, a byproduct of walnut oil production. Rich in proteins, unsaturated fatty acids, minerals, and polyphenols (e.g., ellagic acid, catechins), walnut meal offers potential health benefits, including antioxidant and anti-inflammatory effects [5]. However, in terms of protein composition, soybean meal typically contains 40–50% protein with a balanced amino acid profile, particularly high in lysine [6]. During soy sauce fermentation, proteases secreted by microorganisms like *A. oryzae* efficiently hydrolyze soybean proteins into peptides and free amino acids, contributing to the rich umami flavor of soy sauce [7]. In contrast, walnut meal has a protein content of around 40%, similar to soybean meal, but its amino acid composition is distinct. Walnut protein is rich in arginine and glutamic acid [8], where arginine has positive physiological effects such as vasodilation and immune regulation, while glutamic acid serves as a precursor for the umami compound monosodium glutamate [9]. However, walnut meal is deficient in sulfur-containing amino acids, potentially limiting the synthesis of related flavor compounds. Its proteins are mainly glutelin and prolamins, which resist hydrolysis. Moreover, polyphenols may modify the fermentation microenvironment through protein complexation or enzyme inhibition [10]. Soybean meal contains fermentable sugars that serve as essential energy sources for microbial growth during initial fermentation, critically influencing soy sauce production [11]. Walnut meal, on the other hand, is rich in polysaccharides like cellulose and hemicellulose. These complex polysaccharide structures are difficult to efficiently break down by conventional strains like *A. oryzae* in traditional soy sauce fermentation systems, limiting raw material utilization [12]. These differences result in significant distinctions between WSS and traditional soy sauce in terms of fermentation efficiency and flavor formation mechanisms. However, the dynamic changes in microbial communities and their associations with metabolic products in WSS have yet to be systematically elucidated.

Currently, analyzing the composition and changes in microorganisms in traditional fermented foods plays a vital role in understanding production efficiency, quality characteristics, flavor, and safety [13]. Advances in omics technologies such as metagenomics, transcriptomics, and metabolomics have made research on fermentation techniques and the efficiency and mechanisms of product conversion and synthesis more mature [14]. Microbial omics technologies have revealed that the high expression of protease genes in *A. oryzae* is key to the efficient degradation of proteins in traditional soy sauce fermentation [15]. Additionally, in metabolic networks related to characteristic flavor formation, highly expressed functional genes in microorganisms are primarily associated with carcarbohydrate and amino acid metabolism [16]. However, the unique components of walnut meal in WSS may reshape the microbial community structure. On one hand, polyphenols may inhibit the activity of neutral proteases in *A. oryzae*, affecting protein hydrolysis [17]. Additionally, cellulases secreted by *A. niger* may be more suited for the degradation of polysaccharides in walnut meal [18], but their synergistic or competitive effects with *A. oryzae* remain unclear. Furthermore, the unique “gas–liquid-solid” triphasic microenvironment of solid-state fermentation may exacerbate the spatial heterogeneity of microbial communities, further complicating the fermentation mechanism of WSS.

This study focuses on the solid-state fermentation of walnut meal-soybean meal mixed raw materials. It employs high-throughput sequencing to analyze the effects of different *Aspergillus* strains (*A. oryzae* and *A. niger*) inoculation and fermentation time on microbial community diversity. Combined with molecular docking technology, the study aims to reveal the inhibitory effects of walnut polyphenols on proteases and the interactions between proteases secreted by different *Aspergillus* strains and walnut proteins, while correlating these with changes in physicochemical indicators. The goal is to elucidate the potential microbial driving mechanisms in WSS fermentation. The findings will not only provide theoretical support for optimizing the production of novel soy sauces but also enrich the research on microbial ecology in solid-state fermentation. This study is innovative in clarifying the core functional flora that are essential to the formation of polypeptides. Furthermore, this study reveals the influence of interactions between polyphenol-enzymes on the efficiency of walnut-based soy sauce fermentation, which provides a theoretical basis and an implementation pathway for the efficient utilization of raw materials and the optimization of the process.

## 2. Materials and Methods

### 2.1. Subsection

Walnut meal and soybean meal are provided by Wushi Xianhong Food Brewing Co., Ltd. (Aksu, China), *A. oryzae* 3.042 (Shandong Hezhong Kangyuan Biotechnology Co., Ltd., Zibo, China), and *A. niger* 3.350 (Shandong Hezhong Kangyuan Biotechnology Co., Ltd., Zibo, China) were used. Sodium hydroxide (AR), potassium hydrogen phthalate (≥99.8%), phenolphthalein, 100% ethanol solution, and 37% wt formaldehyde solution were all sourced from Aladdin Chemical Reagent Co., Ltd. (Shanghai, China). The protease activity kit was purchased from Suzhou Grace Biotechnology Co., Ltd. (Suzhou, China).

PHS-3C-02 laboratory pH meter (Zhejiang Airuipu Instrument Co., Ltd., Quzhou, China), constant temperature incubator (Shanghai Huitai Instrument Manufacturing Co., Ltd., Hangzhou, China), microplate reader (Molecular Devices Co., Ltd., Shanghai, China), and high-speed centrifuge (Thermo Fisher Scientific Co., Ltd., Shanghai, China).

### 2.2. Preparation of Walnut-Based Soy Sauce

**Figure 1:**
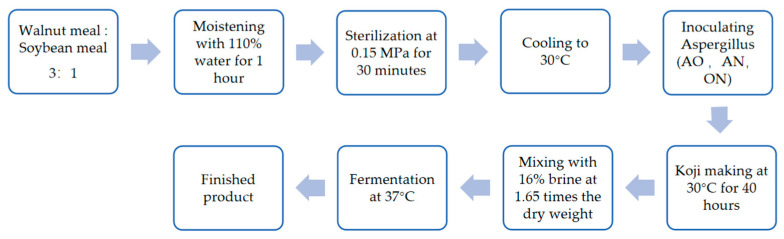
Flowchart of Walnut-based Soy Sauce Fermentation.

The preparation of solid WSS is illustrated in Figure 1. First, the walnut meal was defatted, and then mixed with soybean meal at a precise mass ratio of 3:1. Next, water equivalent to 110% of the dry weight of the mixed raw materials was added for a 1 h moistening process to ensure thorough water absorption, facilitating subsequent treatment. Subsequently, the mixture was sterilized at 0.15 MPa for 30 min to eliminate miscellaneous bacteria under high temperature and pressure while inducing preliminary denaturation of macromolecules in the raw materials. After sterilization, the mixture was cooled to 30 °C and inoculated with *Aspergillus* to ensure microbial activity. The mass ratio of *Aspergillus* to dry substrate is 5 per 10,000. In the ON group, *A. oryzae* and *A. niger* each account for half of the total *Aspergillus* mass.

To evaluate the impacts of different *Aspergillus* strains on the fermentation characteristics of WSS, nine 500 g aliquots of raw materials were randomly assigned to three groups. The groups were inoculated with *A. oryzae* (AO group), *A. niger* (AN group), and a mixture of *A. oryzae* and *A. niger* (ON group), respectively. Post-inoculation, the mixtures were subjected to a 40 h koji-making process at 30 °C, during which *Aspergillus* strains proliferated extensively, secreting enzymes to efficiently decompose proteins and starch in raw materials, while accumulating enzymes and intermediate products. After koji-making, a 16% saline solution (1.65-fold the dry weight of the material) was added to adjust salinity, provide an ionic environment, and inhibit salt-sensitive bacteria. Subsequent soy sauce post-fermentation was conducted at 37 °C, where microorganisms and their enzyme systems utilized pre-formed enzymatic hydrolysis products for further metabolism, generating amino acids, organic acids, and flavor compounds—key factors shaping soy sauce quality. The final product was a preliminary WSS, integrating the flavors of walnuts and soybeans, with distinct nutritional value and taste.

### 2.3. Determination of Physicochemical Properties of Walnut-Based Soy Sauce

The pressed liquid from solid WSS is directly used for pH determination. The total acid content was determined by sodium hydroxide titration. A 5 mL soy sauce sample solution was diluted to a 100 mL volumetric flask and titrated with 0.05 mol/L sodium hydroxide solution until the pH meter indicated 8.2 as the endpoint. The results obtained were expressed as a percentage (% lactic acid). The amino nitrogen content in the samples was determined by titration. A 5 mL soy sauce sample was diluted to 100 mL in a volumetric flask with deoxygenated water. Then, 20 mL of the diluted solution was mixed with 60 mL of deoxygenated water, and 10 mL of 37% wt formaldehyde solution was added. The mixture was titrated with 0.05 mol/L sodium hydroxide solution (calibrated with potassium hydrogen phthalate) until the pH reached 9.2, and the amount of sodium hydroxide consumed was recorded.

Determination of protease activity was performed using the total protease kit from Suzhou Grace Biotechnology Co., Ltd. (Suzhou, China) The method relies on the catalytic hydrolysis of azocasein by total protease, and the resulting product has a light absorption at 366 nm. The total protease activity was determined by measuring the change in absorbance.

### 2.4. Detection of Microbial Diversity in Walnut-Based Soy Sauce

Based on the bacterial 16S rRNA gene and fungal ITS2 gene, high-throughput sequencing technology was used to identify the microbial diversity of soy sauce fermentation samples with different *Aspergillus* species and at different time points, followed by cluster analysis and principal component analysis. The samples were sent to Shanghai Majorbio Bio-pharm Technology Co., Ltd. (Shanghai, China) for Illumina MiSeq high-throughput sequencing of bacterial and fungal diversity [19]. For amplification of the bacterial 16S rRNA gene fragment, universal primers 338F (5′-ACTCCTACGGGAGGCAGAG-3′) and 806R (5′-GGACTACHVGGGTWTCTAAT-3′) were used; for fungi, the amplification primers were ITS1 (5′-TCCGTAGGTGACCTGG-3′) and ITS4 (5′-TCCGTCGTTTAGATGC-3′). Through database comparison, the diversity and relative content of fungal and bacterial microorganisms were analyzed.

### 2.5. Molecular Docking Simulation

The main walnut protein (11S globulin seed storage protein), *A. oryzae* neutral protease, and *A. niger* acid protease were modeled by Swiss-model homology modeling (https://swissmodel.expasy.org/interactive (accessed on 20 July 2025)), and the three-dimensional structures of polyphenols were obtained from PubChem (https://pubchem.ncbi.nlm.nih.gov/). For the preprocessing of receptors and ligands and the docking process, Autodock Tool-1.5.6 (La Jolla, CA, USA) was used [20]. PyMOL 3.1.3 (New York, NY, USA) was employed to visualize the docking results.

### 2.6. Statistical Analysis

Statistical analysis of physicochemical indicators was performed using GraphPad Prism 8 (GraphPad Software Inc., San Diego, CA, USA). Group differences were identified using one-way analysis of variance (ANOVA) followed by Tukey’s multiple comparison posttest. All data were shown as mean ± SD, where a *p*-value of less than 0.05 indicates statistical significance.

Alpha diversity indices such as Chao 1 and Shannon were calculated using mothur software (v1.48.0, developed by the University of Michigan, Ann Arbor, MI, USA; http://www.mothur.org/wiki/Calculators (accessed on 26 June 2025)), and Wilcoxon rank-sum test was performed using GraphPad Prism 8 (v8.4.3, GraphPad Software Inc., San Diego, CA, USA) to analyze differences in Alpha diversity between groups. Principal Coordinate Analysis (PCoA) based on the Bray–Curtis distance algorithm, PERMANOVA non-parametric test (for analyzing significance of microbial community structure differences between groups), Distance-based Redundancy Analysis (db-RDA) (for assessing impacts of physicochemical indicators on bacterial community structure), Linear regression analysis (for evaluating impacts of key physicochemical indicators on microbial Alpha diversity indices), and Spearman correlation analysis (|r| > 0.6, *p* < 0.05; for species selection in correlation network analysis) were all conducted using R software (v4.3.2, R Core Team, Vienna, Austria; https://www.r-project.org/), with the “vegan” package (v2.6-4) for PCoA, PERMANOVA and db-RDA, and the “corrplot” package (v0.92) for Spearman correlation analysis. LEfSe analysis (Linear Discriminant Analysis Effect Size, LDA > 2, *p* < 0.05) was used to identify bacterial taxa with significantly different abundances from phylum to genus levels between groups, implemented via LEfSe software (v1.0, Harvard T.H. Chan School of Public Health, Boston, MA, USA; http://huttenhower.sph.harvard.edu/LEfSe (accessed on 28 June 2025)).

## 3. Results and Discussion

### 3.1. Analysis of Physicochemical Results of WSS

**Figure 2:**
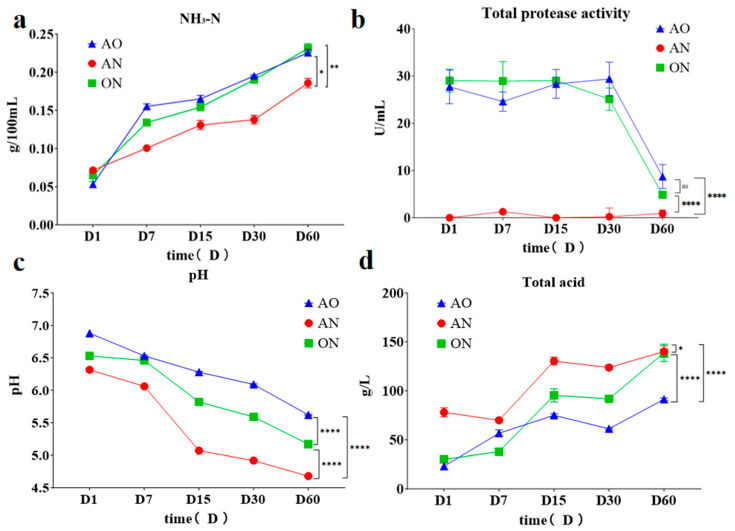
Changes in properties in walnut-based soy sauce fermentation. (**a**) Changes in amino nitrogen content; (**b**) Changes in total protease; (**c**) Changes in pH; (**d**) Changes in total acid content. *, *p* < 0.05; **, *p* < 0.01, ****, *p* < 0.0001, ns, No significant difference (One-way analysis of variance and Tukey’s multiple comparison test) compared among all groups. AO, AN, and ON stands for *A. oryzae*, *A. niger*, and mixed *A. oryzae* and *A. niger*, respectively.

NH_3_-N content, a key quality parameter, reflects protease-mediated protein hydrolysis efficiency. As shown in Figure 2a, the *A. oryzae*-inoculated group demonstrated significantly higher NH_3_-N accumulation rates, indicating enhanced proteolysis. However, WSS NH_3_-N levels remained substantially lower than the traditional soy sauce threshold (≥0.4 g/100 mL). Zhao et al. [12] reported that walnut meal contains lower and structurally more complex protein content (less than half of soybean meal), predominantly composed of harder-to-hydrolyze glutelin and prolamins. The neutral/alkaline proteases from *A. oryzae* show limited hydrolysis efficiency toward these proteins, whereas soybean proteins are mainly more easily hydrolyzable globulins. The most rapid NH_3_-N increase occurred during the initial 7 days of fermentation, after which the accumulation rate progressively decreased.

Figure 2b reveals that the *A. oryzae* group maintained significantly higher protease activity than *A. niger* during the initial 30 fermentation days, followed by a sharp decline (Day 30–60). This pattern reflects progressive depletion of readily hydrolyzable soybean proteins. Meanwhile, walnut flour is rich in polyphenolic compounds such as tannins and tannic acid. It is hypothesized that these compounds can bind to proteins to form insoluble complexes that prevent proteases from entering the cleavage site and inhibit the growth of *A. oryzae*. [21]. Furthermore, Increasing salinity due to water evaporation progressively inhibited microbial and enzymatic activities [22]. Consequently, the observed high protease activity during the initial 30 days drove protein hydrolysis, accounting for the significantly faster NH_3_-N accumulation compared to subsequent phases. Notably, *A. niger* exhibited minimal initial protease activity. This weakness is likely attributable to its proficiency in secreting glycoside hydrolases and organic acids; proteases constitute only a minor fraction of its enzymatic repertoire and are primarily acidic types adapted to a pH range of 2 to 5.0 [23], Throughout the soy sauce fermentation process, the pH remained above the optimal range for these acidic proteases, resulting in persistently weak enzyme activity in the AN group. The inherent physiological characteristics of AN determine that it is not typically considered a highly efficient proteolytic microorganism; thus, it is generally more suitable as an auxiliary culture in soy sauce fermentation.

During soy sauce fermentation, the pH ultimately stabilizes within the range of approximately 4.5 to 5.5. A pH below 4.0 may indicate abnormal fermentation, whereas a pH exceeding 5.5 could suggest insufficient fermentation. The acidification, primarily driven by lactic acid bacteria, is the main cause of pH reduction. Crucially, Soy sauce’s umami and flavor compounds exhibit optimal stability within this mildly acidic environment [23,24,25]. As shown in Figure 2c, post fermentation revealed distinct pH dynamic, the AO group maintained significantly higher pH than the AN group, despite a shared decreasing trend. This divergence stemmed from *A. niger*’s prolific secretion of organic acids, whereas *A. oryzae* primarily generated proteases/amylases with limited acidogenesis. Correspondingly, Figure 2d confirmed the AN group’s substantially higher total acidity (progressively increasing) versus AO and ON groups. It was reported that A. niger could utilize complex carbon sources in both soybean and walnut meals more thoroughly, leading to higher acid production and decreased pH value. However, the acid environment might be not beneficial for increasing NH_3_-N levels by degrading proteins in walnut meal. This might be attributed to the inhibition of protease activities and the growth of protease-producing strains during post-fermentation of WSS [26].

### 3.2. Impact of Fermentation with Different Aspergillus Species on Microbial Diversity

As shown in Table 1, the ACE and Chao values of the AN group were higher than those of the AO and ON groups. This indicates that in the walnut fermentation system involving *A. niger*, the microbial community had a greater total number of species and higher richness of rare species. The Shannon index of the ON group was lower than that of the AN and AO groups, while its Simpson index was relatively higher. This suggests that in the microbial community of the ON group, species richness was lower, the concentration of dominant species was higher, the community was dominated by a few species, and the evenness of species distribution was relatively poor. Among α-diversity indices, a high Shannon index coupled with a low Simpson index indicates that the community contains a large number of species with uniform distribution. Conversely, a low Shannon index together with a high Simpson index implies that the community has fewer species and that dominant species are concentrated.

**Figure 3:**
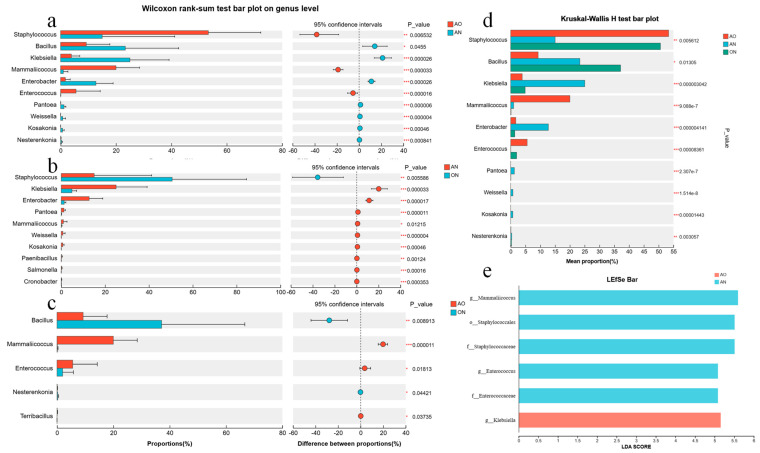
Analysis of species differences among fermentations with different *Aspergillus* species. (**a**) Species differences between the AO and AN groups, (**b**) Species differences between the AN and ON groups, (**c**) Species differences between the AO and ON groups, (**d**) Species differences among the AO, AN, and ON groups, (**e**) LEfSe analysis of WSS inoculated with different *Aspergillus* species. Note: * 0.01 < *p* ≤ 0.05, ** 0.001 < *p* ≤ 0.01, *** *p* ≤ 0.001. Note: AO, AN, and ON stands for *A. oryzae*, *A. niger*, and mixed *A. oryzae* and *A. niger*, respectively.

Figure 3 illustrated the differences in the average relative abundance of the same species among different groups, with annotations indicating whether the differences were significant. It intuitively presented the significance of differences in the same species across multiple groups. In general, as shown in Figure 3d, *Staphylococcus* and *Enterococcus* accounted for a larger proportion in WSS samples supplemented with *A. oryzae*, which were significantly higher than those in the AN group. The proportion of *Bacillus* in the group supplemented with *A. niger* was higher than that in the AO group; meanwhile, the contents of *Klebsiella*, *Enterobacter*, *Pantoea*, *Weissella*, *Kosakonia*, and *Nesterenkonia* in the AN group were all higher than those in the AO and ON groups. The abundance of *Mammaliicoccus* in the AO group was higher than that in the group supplemented with *A. niger*.

As shown in Figure 3, the high abundance of *Staphylococcus* in the AO group may be attributed to the fact that proteolytic products (e.g., short peptides, amino acids) secreted by *A. oryzae* meet their nutritional requirements, and they are potentially involved in the synthesis of soy sauce flavor compounds such as thiols and volatile acids. Liu Hua et al. found that during the brewing of traditional soy sauce, the relative abundance of dominant genera including *Weissella* and *Staphylococcus* remains relatively constant in the late stage of fermentation [27], which is consistent with the results of this study. Kong, F et al. employed three salt-tolerant *Staphylococcus* strains in soy sauce brewing; through analyzing the physicochemical properties, organic acid composition, volatile flavor compounds (VFCs), and sensory characteristics during fermentation, they confirmed that *Staphylococcus* contributes to the acidity, ester aroma, and mellow aroma of soy sauce [28].

*Bacillus* abundance was significantly higher in the *A. niger*-added WWS fermentation system than in the group without *A. niger*. Beyond secreting proteases and amylases, *A. niger* also produces enzymes such as cellulases and pectinases. These additional enzymes hydrolyze polysaccharides present in the raw materials, including cellulose and pectin, into smaller sugar molecules like galacturonic acid and glucuronic acid. This process provides *Bacillus* with a more diverse array of carbon sources. *Bacillus* efficiently utilizes these varied sugars for growth and metabolism. Zheng et al. observed in their research that fermentation with *A. niger* significantly increased the abundance of *Bacillus* [29]. Concurrently, *Bacillus* facilitates the rapid decomposition of starch within the fermentation system, generating organic acids and aromatic compounds such as diacetyl, while also enhancing the antioxidant capacity of the final fermented product [30].

Figure 3e presented the LDA (Linear Discriminant Analysis) scores of different discriminant species, visually demonstrating the relative influence of biomarker taxa identified between groups on the observed differences. The LDA discriminant bar chart statistically identified microbial taxa with significant discriminatory power among multiple groups. Higher LDA scores indicated a greater contribution of the species’ abundance to the observed group differences.

The AO group showed significant enrichment of biomarker taxa including *Mammalicoccus* (Staphylococcaceae), Staphylococcales, *Enterococcus*, and Enterococcaceae. *Mammalicoccus* dominated the *A. oryzae* system, likely due to abundant peptides/carbohydrates from efficient substrate hydrolysis by fungal proteases/amylases. As reported, food-associated *Mammalicoccus* contributes to sensory profiles through carbohydrate/amino acid catabolism and ester synthesis. Flavor-associated small molecular compounds are also generated through aspects of their proteolytic and lipolytic activities [31]. The taxa Staphylococcales and Staphylococcaceae were highly enriched in the AO group, reflecting a microenvironment shaped by *Aspergillus oryzae* that favors the growth of microorganisms within this order and family. Their metabolism produces short-chain fatty acids (SCFAs), such as acetic acid and propionic acid, which contribute to the foundational flavor profile of the sauce (perceived as sour aroma and mellowness) [32]. The AO group exhibited significant enrichment of stress-tolerant *Enterococcus* and Enterococcaceae, compatible with the *A. oryzae* fermentation environment. In contrast, *Klebsiella* was uniquely enriched in the AN group, utilizing polysaccharides/oligosaccharides released by *A. niger* cellulases/pectinases from plant cell walls. Its metabolism produced flavor-active volatiles (e.g., 2,3-butanediol, acetoin), contributing “creamy” and “fruity” notes to the sauce [33].

In general, the fermentation efficiency of WSS under natural fermentation conditions was much lower than that of traditional soybean fermentation. The natural fermentation system had rich microbial diversity, and the growth of *A. oryzae* and *A. niger* might be antagonized by other microorganisms. Meanwhile, the impact of polyphenols in walnut meal on fermentation efficiency could not be ignored. According to Wang Yuzhen et al., the total phenol content in walnut meal reached 2943.12 mg GAE/g, and their study indicated that phenolic acid substances in walnuts (such as ellagic acid, (+)-catechin, chlorogenic acid, and epigallocatechin gallate) all inhibited protease activity [34]. Among them, ellagic acid (EA) had the highest content among polyphenol monomers. Research by Guowan Su et al. showed that polyphenols induced changes in the secondary structure and amino acid composition of walnut protein. These changes led to hindered hydrolysis and enhanced acetylcholinesterase (AChE) inhibition [35], resulting in reduced utilization of walnut protein by microorganisms and thus affecting the efficiency of soy sauce fermentation. High concentrations of polyphenols in walnut meal may directly inhibit the expression of protease genes in *Aspergillus* or bind to the active sites of proteases, altering enzyme conformation and leading to decreased protease activity. The mechanism of interaction between polyphenols and proteases remains to be further studied.

### 3.3. Analysis of the Impact of Different Fermentation Times on Microbial Communities

**Figure 4:**
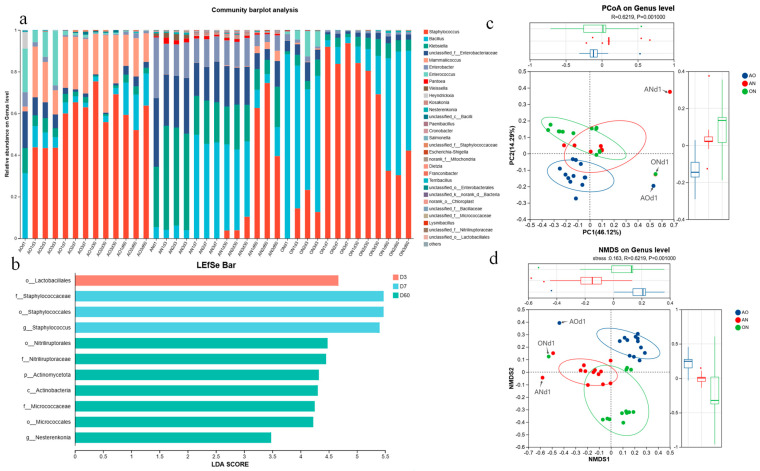
Analysis of the impact of different fermentation times on microbial communities. (**a**) Bacterial community analysis, (**b**) LEfSe analysis at different fermentation periods, (**c**) PCoA analysis of different fermentative *Aspergillus* species. (**d**) NMDS analysis of different fermentative. *Aspergillus* species. Note: AO, AN, and ON stands for *A. oryzae*, *A. niger*, and mixed *A. oryzae* and *A. niger*, respectively.

Microbial composition and abundance varied significantly across fermentation stages and treatments. Figure 4a displays the genus-level bacterial community structure, featuring the top 30 species by relative abundance per sample. The *x*-axis denotes fermentation conditions/durations, while the *y*-axis indicates genus-level relative abundance.

Figure 4 reveals temporally dynamic microbial community structures across all fermentation conditions, with significant intergroup variations in bacterial composition and abundance. While *Staphylococcus* was undetectable at Day 1, its abundance surged during fermentation, peaking at Day 30 in AO/ON groups but delaying until Day 60 in AN group. This pattern can be attributed to substrate availability dynamics. In the early fermentation phase, *A. oryzae* initiates the breakdown of macromolecules in walnut and soybean meal. Nevertheless, limited degradation occurs initially, resulting in insufficient bioavailable nutrients to support substantial *Staphylococcus* growth, hence its low abundance on Day 1. As fermentation progressed, sustained enzymatic activity from *A. oryzae* progressively liberated higher concentrations of small-molecule nutrients. By Day 30, nutrient availability reached levels conducive for rapid *Staphylococcus* proliferation, leading to its peak abundance in AO and ON groups. Conversely, in the AN group, *Staphylococcus* initially faced limited accessible nutrients. Although polysaccharide degradation products accumulated over time, *Staphylococcus* demonstrated relatively low efficiency in utilizing these substrates, requiring extended adaptation periods. Ultimately, by Day 60, more comprehensive substrate decomposition by *A. niger*—yielding higher concentrations of polysaccharide derivatives coupled with sufficient proteolysis-derived nutrients—created optimal conditions for substantial *Staphylococcus* proliferation, resulting in peak abundance.

Throughout the fermentation process, *Bacillus*—the predominant genus in the AO and ON group soy sauces—exhibited an initial decline, reaching its lowest abundance at Day 7, followed by subsequent increase. Conversely, in the AN group, *Bacillus* abundance peaked at Day 7 and gradually decreased thereafter. During early fermentation, *A. oryzae* initiated decomposition of macromolecules in walnut and soybean meals, releasing limited nutrients. At this stage, genera like *Staphylococcus* demonstrated stronger competitive capabilities for these resources, while *Bacillus* was competitively inferior, resulting in its declining abundance. Additionally, evolving environmental parameters further shaped microbial dynamics. Bacillus adapted effectively to the progressively acidic conditions generated by *A. oryzae* metabolism and utilized the enriched nutrient pool in later phases for substantial proliferation, driving its resurgence. In contrast, *A. niger* in the AN group rapidly degraded polysaccharides via pectinases and cellulases, generating abundant sugar derivatives (e.g., glucose, galacturonic acid) early in fermentation. *Bacillus* efficiently assimilated these hydrolysis products, enabling rapid growth and maximal abundance by Day 7. However, the AN group hosted richer distributions of *Klebsiella*, Enterobacteriaceae, and Enterobacter. As fermentation progressed, diminishing nutrients intensified competition. *Bacillus* gradually declined due to competitive disadvantage against these copiotrophic taxa.

Figure 4b highlighted microbial community differences by quantifying the contribution of microbial taxa at various taxonomic levels to inter-group variations through Linear Discriminant Analysis (LDA) scores. During initial fermentation (Day 3), abundant carbohydrates and a near-neutral microenvironment favored Lactobacillales, which rapidly fermented sugars to produce lactic acid. This activity acidified the system, inhibiting pathogenic bacteria growth. By Day 7, the highest LDA scores were observed for family Staphylococcaceae, order Staphylococcales, and genus *Staphylococcus*. At this stage, substantial small-molecule nutrients (e.g., amino acids, monosaccharides) had been liberated through substrate decomposition. *Staphylococcus* populations expanded significantly due to superior nutrient competitiveness and environmental adaptability, establishing these taxa as key discriminators of microbial differences at Day 7. Concurrently, they generated characteristic flavor compounds of soy sauce. Xuefei Shao et al. reported analogous dominance of staphylococci during later fermentation stages in fermented sausages, where they similarly promoted the formation of taste and aroma compounds [36]. At Day 60, taxa including order Nitriliruptorales, family Nitriliruptoraceae, phylum Actinomycetota, and class Actinobacteria exhibited LDA scores approaching 4.5, identifying them as signature microbial groups for this terminal fermentation phase.

As evidenced by Figure 4c, ANOSIM (Analysis of Similarities) revealed significant differences in microbial communities among *Aspergillus* treatment groups (R = 0.6219, *p* = 0.001; *p* < 0.05). This statistically robust distinction demonstrates that *Aspergillus* species substantially altered the microbial composition of the soy sauce fermentation system. The R-value of 0.6219 indicates substantial dissimilarity between groups, signifying that different *Aspergillus* strains exerted distinct effects on microbial communities, ultimately driving divergent community structures. Among the three groups, The AO group exhibited high microbial consistency and structural stability, while the AN group showed greater community discreteness with weaker stability. The ON group demonstrated intermediate stability with moderately conserved community structure. Throughout fermentation, distinct microbial profiles emerged initially across different *Aspergillus* treatments. As fermentation progressed, communities underwent significant restructuring, ultimately converging toward relatively stable configurations in all three groups. Figure 4d shows NMDS results aligned with PCoA outcomes, confirming significant inter-group differences and consistent *Aspergillus*-driven community variation. Furthermore, microbial composition differed significantly between initial (Day 1) and terminal fermentation stages. Both fermentation duration and *Aspergillus* selection significantly influenced walnut sauce microbial dynamics.

### 3.4. Correlation Analysis Results of Physicochemical Properties and Microbial Diversity

**Figure 5:**
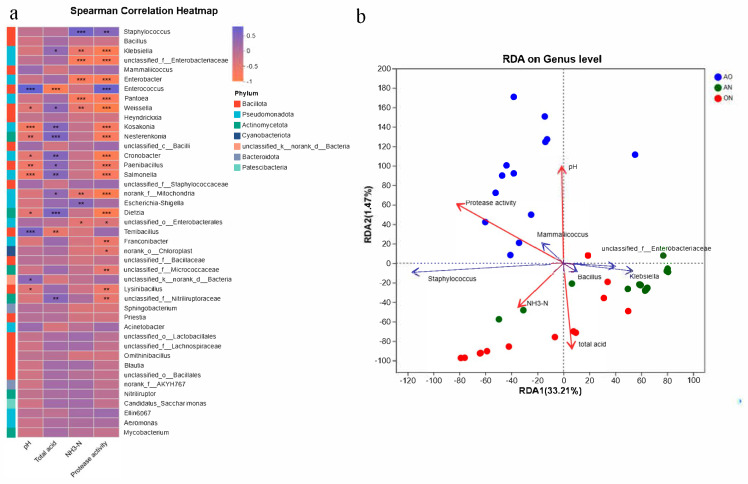
Correlation analysis. (**a**) Correlation heatmap. (**b**) RDA (Redundancy Analysis) scatterplot. Note: * 0.01 < *p* ≤ 0.05, ** 0.001 < *p* ≤ 0.01, *** *p* ≤ 0.001.

Figure 5 illustrated the correlation between microbial taxa and environmental factors, visually presenting both the magnitude and statistical significance of associations between multiple environmental parameters and various species. As shown in Figure 5a, *Enterococcus*, *Terribacillus*, and unidentified *Bacteria* exhibited significantly positive correlations with environmental pH, whereas *Kosakonia*, *Nesterenkonia*, *Cronobacter*, *Paenibacillus*, *Salmonella*, and *Dietzia* showed significantly negative correlations with pH. Conversely, their correlation patterns with total acidity demonstrated an inverse relationship. In fermentation systems, higher total acidity corresponded to lower pH values.

*Enterococcus* and *Terribacillus* exhibited positive correlations with pH, potentially because their enzymatic systems demonstrated higher stability and activity in neutral to slightly alkaline environments [37]. These pH conditions aligned with their physiological requirements, facilitating cellular growth and proliferation, thereby driving positive correlations. Conversely, *Kosakonia* and *Nesterenkonia* showed negative correlations with pH. Such taxa likely possessed acid-adaptation mechanisms, such as specialized proton pumps in cellular membranes that regulated intracellular pH homeostasis. Alternatively, their metabolic pathways (e.g., enzymes involved in anaerobic acid-producing fermentation) might have functioned more actively under acidic conditions. Acidic environments could have provided competitive advantages, consequently increasing their abundance in low-pH settings and thus exhibiting negative correlations.

NH_3_-N represented a crucial quality indicator in soy sauce fermentation. *Staphylococcus* and *Escherichia-Shigella* demonstrated increasing abundance with rising NH_3_-N levels, whereas *Klebsiella*, *Enterobacteriaceae*, *Enterobacter*, *Pantoea*, *Weissella*, and *Mitochondria* exhibited declining populations despite progressive accumulation of NH_3_-N during fermentation. *Staphylococcus* and *Escherichia-Shigella* possessed metabolic adaptations enabling resilience to fermentation environment shifts. As physicochemical parameters (e.g., pH, osmotic pressure) evolved throughout fermentation, these taxa-maintained functionality within tolerance thresholds. The increasing NH_3_-N availability synergized with their metabolic competencies, thereby promoting population expansion. Conversely, negatively correlated taxa likely possessed divergent nitrogen source preferences. They preferentially utilized alternative nitrogen forms rather than competing directly for ammonia nitrogen. When NH_3_-N concentrations increased, these organisms faced competitive exclusion by *Staphylococcus* and *Escherichia-Shigella*, resulting in insufficient nitrogen acquisition to sustain growth and consequent population decline.

Regarding protease activity, *Staphylococcus* and *Enterococcus* exhibited significantly positive correlations with protease activity levels. In contrast, *Klebsiella*, Enterobacteriaceae, *Enterobacter*, *Pantoea*, and *Weissella* demonstrated significantly negative correlations with protease activity. *Staphylococcus* and *Enterococcus* functioned as protease-producing bacteria that secreted extracellular proteases. These proteases might directly decompose fermentation substrates, thereby enhancing overall protease activity in the system. Certain negatively correlated taxa (e.g., *Pantoea*) secreted protease inhibitors or reduced enzymatic activity through intracellular metabolic consumption of environmental free proteases.

RDA analysis (Figure 5b) clearly revealed differences in the associations between microorganisms and physicochemical indices among different fermentation groups (AO/AN/ON). In the AO group, the arrow of *Staphylococcus* highly overlapped with sample points, and there was a small angle (positive correlation) between its arrow and those of protease activity and NH_3_-N. This indicated that *Staphylococcus* was the core taxon in the AO group, which promoted protein decomposition and increased NH_3_-N content via high protease activity, thereby driving fermentation quality. In the AN group, the arrows of *Klebsiella* and *Bacillus* overlapped with sample points; there was a small angle (positive correlation) between their arrows and the pH arrow, and a large angle (negative correlation) with the total acid arrow. That is, these two genera were the core taxa in the AN group, which shaped the fermentation microenvironment by alkalizing the environment and consuming acids. In the ON group, the arrows of Enterobacteriaceae and *Mammaliicoccus* overlapped with sample points; there was a small angle (positive correlation) between their arrows and the total acid arrow, and a large angle (negative correlation) with the pH arrow. This meant that taxa such as Enterobacteriaceae were the core taxa in the ON group, which influenced the fermentation process by producing acids, acidifying the environment, and reducing pH.

The arrow of protease activity was long and pointed to the AO group, indicating that it was mainly driven by *Staphylococcus* in the AO group, and single-strain fermentation with *A. oryzae* was more conducive to protease synthesis and protein decomposition. The small angle between the NH_3_-N content arrow and those of the AO group and *Staphylococcus* showed that its accumulation was directly related to the protein decomposition by *Staphylococcus* in the AO group, and fermentation with *A. oryzae* was the main factor increasing NH_3_-N content. The small angle between the total acid arrow and those of the ON group and Enterobacteriaceae indicated that its accumulation was mainly caused by Enterobacteriaceae in the ON group, and mixed fermentation increased total acid content via synergistic acid production.

### 3.5. Molecular Docking of Protease with Walnut Protein and Walnut Polyphenols

The template used for walnut protein modeling was Q2TPW5.1. A (11S globulin seed storage protein, derived from plants of the genus Juglans). The sequence identity between the target sequence and the template sequence was 100.00%, with a GMQE value of 0.85, indicating that the modeling result was highly reliable. The template used for modeling the neutral protease from *A. oryzae* was P46076.1.A (Neutral protease 2, derived from *A. oryzae*), with a GMQE value of 0.91. This indicated that the model had high reliability and could be used for subsequent structural and functional analyses. The acid protease from *A. niger* was modeled using A0A319DLK0.1.A (Aspergillopepsin, derived from *Aspergillus* ellipticus CBS 70779), with a GMQE value of 0.90, confirming successful modeling.

**Figure 6:**
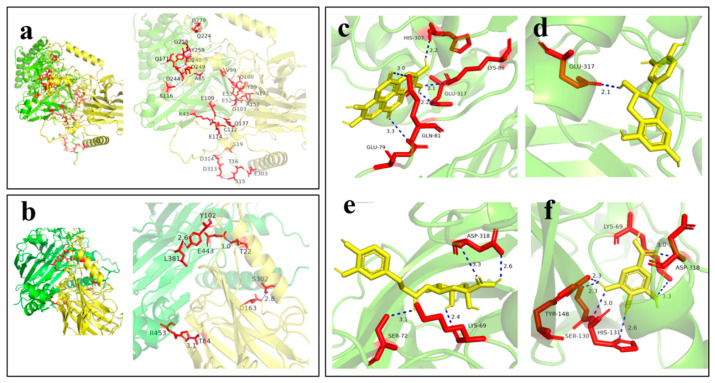
Molecular Docking of Protease with Walnut Protein and Walnut Polyphenols. (**a**) Docking of neutral protease from *A. oryzae* with walnut protein; (**b**) Docking of acid protease from *A. niger* with walnut protein; (**c**) Docking of *A. oryzae* with ellagic acid; (**d**) Docking of *A. oryzae* with catechin; (**e**) Docking of *A. oryzae* with chlorogenic acid; (**f**) Docking of *A. oryzae* with gallic acid. The red sections mainly represent key amino acid residues or ligand molecules involved in the interaction.

Molecular docking results showed that in the docking of neutral protease from *A. oryzae* with walnut protein (Figure 6a), the interaction sites involved numerous amino acids, with interactions distributed widely and forming a complex binding network. This indicated that its binding modes with walnut protein were diverse, and binding was achieved through the synergistic effect of multiple amino acids. The complex binding mode and extensive interaction sites might have caused the structure of walnut protein to be destroyed at multiple points, promoting the gradual hydrolysis of the protein into various small-molecule peptides and amino acids, which was conducive to the diverse production of flavor substances in soy sauce fermentation. For the acid protease from *A. niger* (Figure 6b), the docking involved relatively fewer amino acids, and the binding regions were relatively concentrated. This might have limited its hydrolytic effect on walnut protein, resulting in an overall fermentation rate significantly lower than that of the *A. oryzae* group.

In this study, four relatively abundant polyphenols in walnuts—ellagic acid (EA), catechin (CAT), chlorogenic acid (CA), and gallic acid (GA)—were selected to dock with the neutral protease from *A. oryzae* to investigate their effects on WSS fermentation. As shown in Figure 6c–f, polyphenols bound tightly to the amino acids in the active center of the neutral protease from *A. oryzae* through hydrogen bonds and hydrophobic interactions. The polyphenols occupied the substrate-binding sites in the active center of the protease, directly preventing the binding of walnut protein to the enzyme and inhibiting protein hydrolysis. Meanwhile, the multi-site interactions between polyphenols and the enzyme might have altered the conformation of the enzyme’s active center, reduced its catalytic efficiency, and decreased the production of flavor precursors such as amino acids and peptides.

Molecular docking results showed that there were differences in binding energies between four polyphenols (ellagic acid, catechin, chlorogenic acid, and gallic acid) and the neutral protease from *A. oryzae* (AO). The binding energy data (Table 2) indicated that the absolute value of the binding energy for AO-CA was the largest overall; for example, the data in Group 1 reached −8.27 kcal/mol, suggesting that its binding to the protease was more stable. The average binding energy for AO-EA was also relatively high, indicating strong binding stability. From the docking diagrams, taking AO-EA as an example (Figure 6a), ellagic acid formed multiple sets of hydrogen bonds with amino acid residues such as HIS-307, LYS-86, and GLU-317 of the protease (e.g., a 2.2 Å hydrogen bond between HIS-307 and ellagic acid). These hydrogen bond interactions enabled the polyphenol to bind to the active center region of the protease, hindering the binding of the substrate to the enzyme. In AO-CA (Figure 6c), chlorogenic acid formed hydrogen bonds with amino acids such as ASP-318 and LYS-69, which also generated steric hindrance to the active center of the protease. In summary, different polyphenols stably bound to the neutral protease from *A. oryzae* through interactions such as hydrogen bonds formed with amino acid residues in the enzyme’s active center. The lower the binding energy, the more stable the binding, which more easily hindered the catalysis of the substrate by the protease, affected the hydrolysis of proteins during *A. oryzae* fermentation, and thus might have altered the fermentation quality and efficiency of WSS.

In addition, Cheng et al. used molecular docking results in their research to reveal the mechanism by which polyphenols inhibit the activity of glycosidases and proteases through stable binding to the active centers of the enzymes. This inhibitory effect indirectly inhibited fungal growth and protease secretion by reducing nutrient supply to fungi [38]. Makarewicz et al. pointed out that polyphenols could bind to the active centers of microorganism-secreted enzymes (e.g., proteases, glycosidases), altering enzyme conformation and inhibiting their activity. Meanwhile, they could also affect community structure and metabolic functions by selectively inhibiting or promoting the growth of specific microorganisms [39]. This mechanism provided a theoretical basis for analyzing the inhibition of *A. oryzae*/*A. niger* protease activity by walnut polyphenols and the regulation of dynamic changes in microbial communities during WSS fermentation. Specifically, polyphenols in walnuts might have affected the catalytic efficiency of enzymes and the growth metabolism of microorganisms in the fermentation system through similar modes of action, thereby altering the fermentation process and quality characteristics of soy sauce.

## 4. Conclusions

This study explored the link between microbial community dynamics and fermentation traits in WSS, aiming to clarify its fermentation mechanisms. Combined with physicochemical monitoring, high-throughput sequencing, and molecular docking, it was revealed that *A. oryzae* and *A. niger* shaped distinct fermentation microenvironments. Specifically, AO promoted protein hydrolysis via high neutral protease activity and facilitated faster NH_3_-N accumulation. In addition, AO enriched protease-producing species during post-fermentation of WSS, which showed positive correlations with NH_3_-N production. Additionally, walnut polyphenols might inhibit activity of AO-sourced neutral proteases based on molecular docking This research provides a theoretical basis for optimizing WSS production and enriches understanding of microbial ecology in solid-state fermentation. Future work may focus on substrate ratio optimization, screening of polyphenol-tolerant strains, and multi-omics-based elucidation of metabolic pathways to achieve targeted fermentation control.

## Figures and Tables

**Table 1:** Alpha diversity index of fermented soy sauces.

Sample/Estimators	Starters	Post-Fermentation Time (d)	ACE	Chao	Shannon	Simpson	Coverage
AOd1	AO	1	14	14	1.783	0.199	1
AO1d3	AO	3	0	10	1.563	0.274	1
AO2d3	AO	3	14.75	14	1.645	0.257	0.99995
AO3d3	AO	3	10	10	1.544	0.28	1
AO1d7	AO	7	9	9	1.181	0.425	1
AO2d7	AO	7	0	10	1.021	0.492	1
AO3d7	AO	7	0	10	1.094	0.466	1
AO1d30	AO	30	10	10	0.777	0.606	1
AO2d30	AO	30	10	10	1	0.451	1
AO3d30	AO	30	10	10	0.917	0.533	1
AO1d60	AO	60	13	13	1.256	0.401	1
AO2d60	AO	60	13	13	1.403	0.337	1
AO3d60	AO	60	11	11	1.129	0.45	1
ANd1	AN	1	12	12	1.419	0.28	1
AN1d3	AN	3	18	18	1.393	0.334	1
AN2d3	AN	3	20	20	1.459	0.31	1
AN3d3	AN	3	17	17	1.415	0.317	1
AN1d7	AN	7	20	20	1.432	0.32	1
AN2d7	AN	7	22	22	1.481	0.292	1
AN3d7	AN	7	19	19	1.426	0.302	1
AN1d30	AN	30	20	20	1.644	0.257	1
AN2d30	AN	30	20	20	1.686	0.245	1
AN3d30	AN	30	20	20	1.71	0.224	1
AN1d60	AN	60	17	17	1.331	0.419	1
AN2d60	AN	60	14	14	1.039	0.568	1
AN3d60	AN	60	20	20	1.706	0.236	1
ONd1	ON	1	9	9	0.528	0.785	1
ON1d3	ON	3	0	9	1.095	0.498	1
ON2d3	ON	3	11	11	1.431	0.312	1
ON3d3	ON	3	0	10	1.182	0.457	1
ON1d7	ON	7	10.581	10	0.386	0.848	0.999951
ON2d7	ON	7	8	8	0.646	0.711	1
ON3d7	ON	7	0	7	0.319	0.879	1
ON1d30	ON	30	0	8	0.631	0.717	1
ON2d30	ON	30	10	10	0.735	0.661	1
ON3d30	ON	30	0	7	0.943	0.52	1
ON1d60	ON	60	14.534	14	1.145	0.404	0.999951
ON2d60	ON	60	10	10	1.035	0.448	1
ON3d60	ON	60	11	11	1.074	0.411	1

Note: AO, AN, and ON stand for *A. oryzae*, *A. niger*, and mixed *A. oryzae* and *A. niger*, respectively. AO1d3 represents the first set of samples from the third day of the *A. oryzae* parallel trio, AN1d3 represents the first set of samples from the third day of the *A. niger* group, and ON1d3 represents the first set of samples from the third day of the *A. oryzae* and *A. niger* mixed group. And so on.

**Table 2:** Binding energy between polyphenols and proteases.

No.	AO-GA (kcal/mol)	AO-EA (kcal/mol)	AO-CA (kcal/mol)	AO-CAT (kcal/mol)
1	−6.69	−7.41	−8.27	−7.22
2	−6.63	−7.40	−7.57	−6.95
3	−6.58	−7.36	−7.41	−6.87
4	−6.57	−7.31	−7.34	−6.78
5	−6.47	−7.17	−7.24	−6.63
6	−6.34	−7.14	−7.06	−6.54
7	−6.12	−7.12	−6.99	−6.48
8	−5.76	−7.10	−6.72	−6.36
9	−5.75	−7.07	−6.72	−6.28
10	−5.74	−6.63	−6.51	−6.13

## Data Availability

The original contributions presented in this study are included in the article. Further inquiries can be directed to the corresponding author.

## Abbreviations

The following abbreviations are used in this manuscript:

WSS	Walnut-based Soy Sauce
AO	*Aspergillus oryzae*
AN	*Aspergillus niger*
ON	*Aspergillus oryzae* and *Aspergillus niger*
EA	ellagic acid
CAT	catechin
CA	chlorogenic acid
GA	gallic acid
